# Triple therapy for COPD: a crude analysis from a systematic review of the evidence

**DOI:** 10.1177/1753466619885522

**Published:** 2019-11-06

**Authors:** Jose Luis Lopez-Campos, Laura Carrasco-Hernandez, Esther Quintana-Gallego, Carmen Calero-Acuña, Eduardo Márquez-Martín, Francisco Ortega-Ruiz, Joan B. Soriano

**Affiliations:** Instituto de Biomedicina de Sevilla (IBiS), Unidad Médico-Quirúrgica de Enfermedades Respiratorias, Hospital Universitario Virgen del Rocío/Universidad de Sevilla, Avda. Manuel Siurot, s/n., Seville, 41013, Spain; Centro de Investigación Biomédica en Red de Enfermedades Respiratorias (CIBERES). Instituto de Salud Carlos III, Madrid, Spain; IBiS, Unidad Médico-Quirúrgica de Enfermedades Respiratorias, Hospital Universitario Virgen del Rocío/Universidad de Sevilla, Spain; CIBERES, Instituto de Salud Carlos III, Madrid, Spain; IBiS, Unidad Médico-Quirúrgica de Enfermedades Respiratorias, Hospital Universitario Virgen del Rocío/Universidad de Sevilla, Spain; CIBERES, Instituto de Salud Carlos III, Madrid, Spain; IBiS, Unidad Médico-Quirúrgica de Enfermedades Respiratorias, Hospital Universitario Virgen del Rocío/Universidad de Sevilla, Spain; CIBERES, Instituto de Salud Carlos III, Madrid, Spain; IBiS, Unidad Médico-Quirúrgica de Enfermedades Respiratorias, Hospital Universitario Virgen del Rocío/Universidad de Sevilla, Spain; CIBERES, Instituto de Salud Carlos III, Madrid, Spain; IBiS, Unidad Médico-Quirúrgica de Enfermedades Respiratorias, Hospital Universitario Virgen del Rocío/Universidad de Sevilla, Spain; CIBERES, Instituto de Salud Carlos III, Madrid, Spain; CIBERES, Instituto de Salud Carlos III, Madrid, Spain; Hospital Universitario de la Princesa (IISP), Universidad Autónoma de Madrid, Madrid, España

**Keywords:** clinical trials, COPD, systematic review, triple therapies

## Abstract

We systematically reviewed the current knowledge on fixed-dose triple therapies for the treatment of chronic obstructive pulmonary disease (COPD), with a specific focus on its efficacy *versus* single bronchodilation, double fixed dose combinations, and open triple therapies. Articles were retrieved from PubMed, Embase, and Scopus up to 3 August 2018. We selected articles with randomized controlled or crossover design conducted in patients with COPD and published as full-length articles or scientific letters, evaluating triple therapy combinations in a single or different inhaler, and with efficacy data *versus* monocomponents, double combinations, or open triple therapies. Our systematic search reported 108 articles, of which 24 trials were finally selected for the analysis. A total of 7 studies with fixed dose triple therapy combinations, and 17 studies with open triple therapies combinations. Triple therapy showed improvements in lung function [trough forced expiratory volume (FEV_1)_ ranging from not significant (NS) to 147 ml], health status using the St. George’s Respiratory Questionnaire [(SGRQ) from NS to 8.8 points], and exacerbations [risk ratio (RR) from NS to 0.59 for all exacerbations] *versus* single or double therapies with a variability in the response, depending the specific combination, and the comparison group. The proportion of adverse effects was similar between study groups, the exception being the increase in pneumonia for some inhaled corticosteroid (ICS) containing groups.

*The reviews of this paper are available via the supplementary material section.*

## Introduction

The availability of the so-called inhaled triple therapy, that is, the combination of an inhaled long-acting ß_2_ agonist (LABA), an inhaled long-acting muscarinic antagonist (LAMA) and an inhaled corticosteroid (ICS) in a single inhalation device, for the treatment of chronic obstructive pulmonary disease (COPD) has been a recent therapeutic novelty. The different clinical trials available demonstrate the efficacy and safety profile of these fixed dose combinations at various stages of clinical development.

Of note, the implementation of a new potential strategy for the treatment of COPD may represent a challenge for the clinician within the step-up or step-down treatment recommendations in response to current guidelines.^1,2^ In addition, the potential risks of over prescribing more intense therapies in a single inhaler may also lead to overtreatment.^3^ Therefore, a global view on the efficacy of this new form of treatment is required to allow the clinical evaluation of these fixed dose combinations (FDC) triple therapies. Specifically, in the current situation where there are considerable prescriptions of open triple therapy for COPD in clinical practice^4–6^ and there are no direct comparative studies between triple therapies FDC.

In this regard, there are at least three recent meta-analyses evaluating the efficacy endpoints of triple therapies combining the results into one single analysis.^7–9^ These meta-analyses have provided valuable information allowing us to have a global view on the efficacy of triple therapies in the management of COPD. However, they either evaluate specific comparisons with some double combinations or single therapies separately, are focused only on few endpoints, combine the results of FDC with open triple therapies, or are focused on efficacy rather than safety. In addition, as in any other research study, meta-analyses may also have critical issues including the identification and selection of studies, the heterogeneity of results, the availability of information, and the analysis of the data. These caveats in performing and interpreting meta-analyses can yield misleading information.^10^ In this situation, the description of raw data on efficacy and safety of FDC triple therapies in a systematic way would provide the clinician a joint global view on the efficacy and safety profiles for each combination complementing the information provided by recent meta-analyses.

Therefore, our objective was to systematically review the current knowledge and summarize raw data about triple therapy for the treatment of COPD, focusing on its efficacy against monotherapies, double therapies, and open triple therapies in terms of lung function, symptoms, and exacerbations. In addition, we also explored the effects on mortality and safety. Although direct comparisons were not possible using the present design, an evaluation of the average improvements of the different clinical efficacy results will help physicians to better understand of the magnitude of the clinical benefits and to evaluate the expected benefits in the patients, finally helping clinical decision making.

## Methods

The present analysis was a systematic review of clinical trials evaluating triple inhaled therapies. A systematic search was performed on 3 August 2018, in PubMed, EMBASE, and Scopus searching for articles evaluating triple therapy combinations, including all drugs marketed in Europe for the treatment of COPD. This search was updated on 7 September 2019 for the combination of glycopyrronium bromide (GB), formoterol fumarate (FOR), and budesonide (BUD). All identified abstracts were retrieved and evaluated. The selection criteria included: randomized controlled or crossover design, conducted in patients with COPD, language restricted to English, evaluating triple therapy combinations in a single or different inhalers, reporting on lung function, respiratory symptoms, or exacerbations *versus* mono-components, double combinations or open triple therapy, and published as full-length articles or scientific letters. We excluded the following trials: studies available only in a congress abstract form, studies which were not original clinical research (i.e. systematic or narrative reviews), and studies reporting subgroup analyses from previous trials.

Upon selection of all studies, information on lung function, symptoms, and exacerbations were recovered. The analysis of the outcome data was carried out on the results reported at the last visit at the end of each trial and in the intention-to-treat population. Lung function parameters analyzed included trough (morning pre-dose) forced vital capacity (FVC), trough forced expiratory volume in one second (FEV_1_) expressed as ml and the number of patients improving at least 100 ml [considered the minimum clinically important difference (MCID)] expressed as percentage or odds ratio (OR), FEV_1_ 5 min post morning dose (as a measure of the rapid onset of action), peak FEV_1_ (defined as the highest FEV_1_ after morning dose), and FEV_1_ area under the curve from 0 to 24 h post morning dose (FEV_1_ AUC_0-24_). Results of lung volumes were also noted in ml by recording total lung capacity, residual volume, forced residual capacity, and inspiratory capacity (IC).

Disease impact was evaluated by symptoms perception including the following variables: dyspnea measured by the transitional dyspnea index (TDI), evaluating the mean improvements and the percentage of patients who showed an improvement of at least 1 TDI point (which is considered the MCID),^11^ expressed as percentage or OR; health-related quality of life as measured by the St. George’s Respiratory Questionnaire (SGRQ), also evaluating the mean improvements and the percentage of patients who showed an improvement in the MCID (4 points in the questionnaire,^12^ expressed as percentage or OR); and rescue medications, evalutated in puffs per day over a 24-h period and as percentage of days with no rescue medication use.

Exacerbations were also included in the analysis. In particular, both the annualized rate ratios of the number of exacerbations expressed as risk ratios (RR) and the time to the first exacerbation expressed as hazard ratios (HR) were evaluated. The analysis focused on all exacerbations, and for moderate-to-severe exacerbations separately.

All of the collected efficacy data were summarized in a Microsoft Excel (Microsoft Corporation, WA, USA) spreadsheet. The mean values at the end of the trial for the intention-to-treat population were collected for each endpoint and presented in tables. We explored different comparisons for triple FDCs *versus* LAMA, triple FDC *versus* LABA, triple FDC *versus* LABA/ICS, triple FDC *versus* LABA/LAMA, and triple FDC *versus* open triple therapies. With this information, we constructed tables where the maximum and minimum significant mean improvements observed in the different trials were presented for all endpoints. If no significant differences were found in a trial, it was registered as the minimum mean improvement and noted as not significant (NS). If this was true for all trials, it was noted as NS. Because patient-based data were not available, we did not carry out any analysis on the direct comparison of results that were not a specific focus of our study. Our aim was to provide a general summary and information on the crude average values of the different triple therapies, with the aim of enabling their clinical evaluation.

## Results

### Study selection

**Figure 1.**
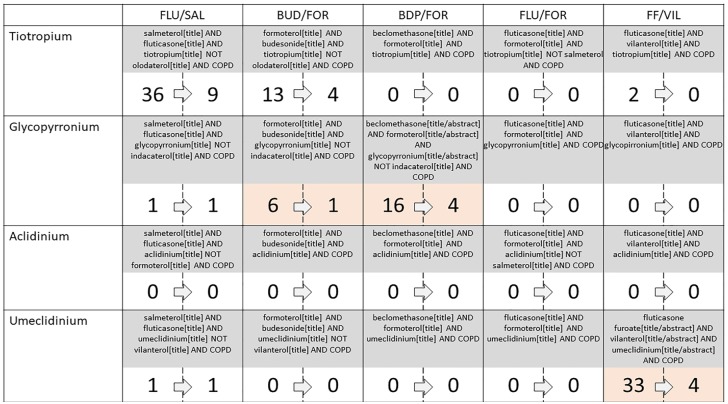
Identification and selection of studies combining triple therapies. Within each combination the number of studies initially identified is referred on the left and the number of studies finally included in the analysis is on the right. Light red highlights combinations including at least one FDC therapy study.

The systematic search reported that 108 articles fulfilled the prespecified search (Figure 1). After the evaluation of the inclusion and exclusion criteria, 84 articles were excluded. The reasons for excluding these were, 59 studies did not have a randomized controlled or crossover design, 30 did not report clinical outcomes in COPD, 23 studies did not evaluate triple therapies, 2 studies reported subgroup analyses, and 1 study was written in Chinese. The final number of studies included was 24, of which 17 evaluated open triple therapies and 7 evaluated FDC triple therapies.

### Triple FDC studies description

In total three different trials were identified for the FDC of GB, FOR, and beclomethasone dipropionate (BDP). In brief, the TRILOGY trial randomized 1367 patients to compare fixed triple combination with BDP/FOR with the primary objectives being pre-dose FEV_1_,

2-h post-dose FEV_1_, and TDI, all of them at week 26, although the study was 52 weeks long.^13^ TRINITY randomized 2690 patients to compare fixed triple combination with tiotropium alone or an open triple combination of BDP/FOR and tiotropium with the primary objectives being annualized moderate-severe exacerbation rate.^14^ Finally, TRIBUTE randomized 2690 patients to compare fixed triple combination with indacaterol/GB fixed dose combination with the dose of 110/50 once daily with the primary objective being annualized moderate-severe exacerbation rate.^15^

In total three different trials were identified for the FDC of umeclidinium bromide (UMEC), vilanterol trifenatate (VI), and fluticasone furoate (FF). In brief, the FULFIL trial randomized 1810 patients to compare fixed triple combination with BUD and FOR with the change from baseline in trough FEV_1_ and in SGRQ total score at week 24, as co-primary endpoints.^16^ In this study, a subset of the first 430 patients to enroll in the trial and consent to longer-term treatment remained on blinded study treatment for up to 52 weeks. The study by Bremner and colleagues randomized 1055 patients with a noninferiority design to compare FDC triple therapy with open triple therapy with FF/VI and UMEC in two separate Ellipta (Glaxosmithkline, Brentford, UK) inhalers, with the primary endpoint defined as the change from baseline in trough FEV_1_ at week 24.^17^ Finally, the IMPACT trial randomized 10,355 patients to compare triple FDCs with FF/VI and with double bronchodilation with UMEC/VI, with the annual rate of moderate or severe COPD exacerbations during treatment as the primary endpoint.^18^

We also identified one study that evaluated a FOR/BUD/GB combination presented in a metered-dose inhaler (MDI). The KRONOS trial randomized 1902 patients to compare this triple FDC with FOR/GB in MDI, with FDC of BUD/FOR in MDI, and the open-label BUD/FOR in a dry powder inhaler. Primary and secondary endpoints and treatment comparisons of interest differed according to regulatory registration requirements between Europe, Canada, and the USA and included FEV_1_ AUC_0-4_ *versus* the LABA/ICS combination and trough FEV_1_ *versus* the LABA/LAMA combination as primary endpoints.^19^ The KRONOS study reported the majority of results over 24 weeks instead of at 24 weeks. Therefore, many results were not available at the same timepoint as other FDC trials and, therefore, were not included in the main tables. In addition, a strong control of the type I error rate was maintained in the analysis of the KRONOS study. In this study, a difference was termed as nominally significant when *p* < 0.05 but not statistically significant after type I error control, or not included in the type I error control strategy.

### Open triple therapies studies description

The description of the different designs and patient’s characteristics of all 17 open triple therapies studies are summarized in the online supplementary Tables S1 and S2. None of the studies reported the blood eosinophils count as was carried out in the FDC studies. Exacerbations in the previous year of the trial were also rarely reported and these were mostly nonfrequent exacerbator patients. The rest of the recorded variables were compatible with including patients with moderate-to-severe lung function impairment.

### Triple therapy *versus* LAMA

The summary of the efficacy findings comparing triple therapies *versus* a LAMA are summarized in Table 1. The only FDC triple study available (TRINITY) met the primary endpoint (moderate-to-severe COPD exacerbation rate).^14^ There were no studies showing results of FF/UMEC/VI combination *versus* a LAMA. Only one study reported efficacy results on endurance time or with endurance shuttle walking test, showing no significant differences between triple and LAMA therapies.^20^

**Table 1.** Summary of the efficacy results of triple therapy *versus* LAMA.

		BDP/FOR/GB	Open triples
Lung function	Trough FVC (ml)	–	NS to 200 (48, 347)
	Trough FEV_1_ (ml)	61 (37, 86)	NS to 210 (109, 315)
	Trough FEV_1_ ⩾100 mL (OR)	1.62 (1.35, 1.95)	–
	FEV_1_ 5 min post morning dose (ml)	–	123 (not reported)
	Peak FEV_1_ (ml)	–	–
	FEV_1_ AUC_0-24_	–	–
	Total lung capacity (ml)	–	NS
	Forced residual capacity (ml)	–	NS
	Residual volume (ml)	–	NS to 930 (875, 991)
	IC (ml)	–*	NS to 1080 (1019, 1150)
Symptoms	Dyspnea (TDI)	–	NS to 2.2 (0.8, 3.5)
	TDI increase ⩾1 point (OR)	–	–
	HRQL (SGRQ)	–*	NS to −8.8 (−6.5, −11.2)
	SGRQ increase ⩾4 points (%)	–	NS to 13.4 (not reported)
	SGRQ increase ⩾4 points (OR)	1.33 (1.11, 1.59)	–
	Rescue medication (puffs/day)	–0.61 (–0.78, –0.44)	NS to −0.67 (−0.44, −0.90)
	Rescue medication (days without)	8.78 (5.74, 11.81)	–
Exacerbations	Number of all exacerbations (RR)	–	NS to 0.59 (0.42, 0.84)
	Time to first exacerbation, all (HR)	–	0.61 (0.41, 0.92)
	Number of moderate-to-severe exacerbations (RR)	0.80 (0.69, 0.92)	0.38 (0.2, 0.57)
	Time to first moderate-to-severe exacerbation (HR)	0.84 (0.72, 0.97)	–

Results expressed as point estimates with 95% CI in parentheses when reported

BDP/FOR/GB, fixed dose combination of beclomethasone, formoterol, and glycopyrronium; FEV_1_, forced expiratory volume in the first second; FF/UMEC/VI, fixed dose combination of fluticasone furoate, umeclidinium, and vilanterol; FEC, forced expired capacity; FVC, forced vital capacity; HR, hazard ratio; HRQL, health-related quality of life; IC, inspiratory capacity; LAMA, long-acting muscarinic antagonist; NS, not significant; OR, odds ratio; RR, risk ratio; SGRQ, St. George’s Respiratory Questionnaire; TDI, transitional dyspnea index. *The original article reported a significant association but provided no numerical data.

### Triple therapy *versus* LABA

Only one study reported results on the comparison of open triple therapy *versus* a LABA.^21^ This study aimed to assess the effects of tiotropium, salmeterol and salmeterol/fluticasone and open triple on airway dimensions in COPD and clinical outcomes were secondary data. The study showed a significant increase favoring the triple combination in 44 ml of trough FVC, 11 ml in trough FEV_1_, and 441 ml in IC, but with no differences in health-related quality of life as measured by the SGRQ. No studies on triple FDC reported comparisons *versus* a LABA.

### Triple therapy *versus* LABA/LAMA

The summary of the findings comparing triple therapies *versus* a LABA/LAMA are summarized in Table 2. All FDC triple studies available met their primary endpoints (annual rate of moderate-to-severe COPD exacerbations).^15,18,19^ Only one study evaluating open triple *versus* LABA/LAMA was identified.^22^ In addition, the combination FF/UMEC/VI showed a significant improvement in mortality from any cause in the IMPACT trial.^18^ The HR ratio for triple therapy *versus* UMEC/VI was 0.58 (CI 95% 0.38–0.88). This mortality analysis was reported as an exploratory analysis not included in the primary or secondary objectives of the trial, for the prespecified on treatment population, with no adjustment for multiplicity, and with an unadjusted *p* value of 0.01. In the KRONOS trial, results were reported over 24 weeks, with significant differences in trough FEV_1_ 22 (4–39) ml, SGRQ 1.22 (–2.30 to ‒0.15), but not in dyspnea by the TDI score or rescue medication.

**Table 2.** Summary of the efficacy results of triple therapy *versus* LABA/LAMA.

		BDP/FOR/GB	FF/UMEC/VI	BUD/FOR/GB	Open triples
Lung function	Trough FVC (ml)	NS	–	–	–
	Trough FEV_1_ (ml)	NS	54 (39, 69)	NS	NS
	Trough FEV_1_ ⩾100 ml (OR)	NS	–	–	–
	FEV_1_ 5 min post morning dose (ml)	–	–	–	–
	Peak FEV_1_ (ml)	–	–	–	–
	FEV_1_ AUC_0-24_	–	–	–	–
	Total lung capacity (ml)	–	–	–	–
	Forced residual capacity (ml)	–	–	–	–
	Residual volume (ml)	–	–	–	–
	IC (ml)	–	–	–	–
Symptoms	Dyspnea (TDI)	–	–	–	–
	TDI increase ⩾1 point (OR)	–	1.33 (1.13, 1.57)	–	–
	HRQL (SGRQ)	−1.68 (not reported)	−1.8 (–2.4, –1.1)	–	NS
	SGRQ increase ⩾4 points (%)	–	–	–	–
	SGRQ increase ⩾4 points (OR)	NS	1.41 (1.29, 1.55)	1.28 (1.01, 1.61)*	–
	Rescue medication (puffs/day)	NS	–	NS	–
	Rescue medication (days without)	NS	–	–	–
Exacerbations	Number of all exacerbations (RR)	–	–	–	NS
	Time to first exacerbation, all (HR)	–	–	–	–
	Number of moderate-to-severe exacerbations (RR)	0.84 (0.72, 0.99)	0.75 (0.70, 0.81)	0.48 (0.37, 0.64)	–
	Time to first moderate-to-severe exacerbation (HR)	NS	0.84 (0.79, 0.89)	0.59 (not reported)	–

Results expressed as point estimates with 95% CI in parentheses when reported. *p value of 0.04, but referred to as nominally significant which denotes *p* < 0.05 but not statistically significant after type I error control or not included in the type I error control strategy.^19^

BDP/FOR/GB, fixed dose combination of beclomethasone, formoterol, and glycopyrronium; FEV_1_, forced expiratory volume in the first second; FF/UMEC/VI, fixed dose combination of fluticasone furoate, umeclidinium, and vilanterol; FVC, forced vital capacity; HR, hazard ratio; HRQL, health-related quality of life; IC, inspiratory capacity; LABA, inhaled long-acting ß_2_ agonist; LAMA, long-acting muscarinic antagonist; NS, not significant; OR, odds ratio; RR, risk ratio; SGRQ, St. George’s Respiratory Questionnaire; TDI, transitional dyspnea index.

### Triple therapy *versus* LABA/ICS

The summary of the findings comparing triple therapies *versus* a LABA/ICS are presented in Table 3. KRONOS^19^ and both FF/UMEC/VI FDC triple studies met their primary endpoints (FULFIL: trough FEV_1_, and SGRQ at week 24;^16^ IMPACT: moderate-severe exacerbations annual rate^18^). The TRILOGY trial identified three primary endpoints and only met two of them (trough FEV_1_ and FEV_1_ 2 hours post-dose), but not dyspnea at week 26.^13^ In the KRONOS trial, results were reported over 24 weeks, with significant differences in trough FEV_1_ 74 (52–95) ml, but not in dyspnea by the TDI score, SGRQ, or rescue medication.

**Table 3.** Summary of the efficacy results of triple therapy *versus* LABA/ICS.

		BDP/FOR/GB	FF/UMEC/VI	BUD/FOR/GB	Open triples
Lung function	Trough FVC (ml)	–	–	–	NS to 243 (178, 308)
	Trough FEV_1_ (mL)	63 (32, 94)	97 (85, 109) to 171 (148, 194)	74 (47, 102)^‡^	NS to 147
	Trough FEV_1_ ⩾100 ml (OR)	2.06 (1.62, 2.62)	4.03 (3.27, 4.97)	–	4.1 to 5.6
	FEV_1_ 5 min post morning dose (ml)	–	–	–	–
	Peak FEV_1_ (ml)	–	–	–	90 (not reported) to 186 (145, 226)
	FEV_1_ AUC_0-24_	–	–	–	–
	Total lung capacity (ml)	–	–	–	NS to 105 (12, 221)$
	Forced residual capacity (ml)	–	–	–	NS
	Residual volume (ml)	–	–	–	NS to 189 (46, 332) $
	IC (ml)	–	–	–	NS to 58 (not reported)
Symptoms	Dyspnea (TDI)	NS	–	–	NS
	TDI increase ⩾1 point (OR)	NS	1.36 (1.19, 1.55)	–	–
	HRQL (SGRQ)	−1.69 (–3.20, –0.17)	−1.8 (−2.4, −1.1) to −2.2 (−1.0, −3.5)	–	NS to −2.16 (−0.49, −3.83)
	SGRQ increase ⩾4 points (%)	–	–	–	–
	SGRQ increase ⩾4 points (OR)	1.33 (1.06, 1.66)	1.41 (1.29, 1.55) to 1.41 (1.16, 1.70)*	NS	NS to 2.01 (1.28, 3.14)
	Rescue medication (puffs/day)	NS	–	NS	NS to −0.72 (−1.08, −0.34)
	Rescue medication (% days without)	NS	–	–	NS to 8.1 (3.6, 12.6)
Exacerbations	Number of all exacerbations (RR)	–	0.65	–	–
	Time to first exacerbation, all (HR)	–		–	–
	Number of moderate-to-severe exacerbations (RR)	0.77 (0.65, 0.92)	0.65 (0.49, 0.86) to 0.85 (0.80, 0.90)	NS	NS
	Time to first moderate-to-severe exacerbation (HR)	0.80 (0.67, 0.97)	0.85 (0.80, 0.90)	NS	–

Results expressed as point estimates with 95% CI in parentheses when reported. *Both trials FULFIL and IMPACT reported the same point estimates with different confidence intervals. $97.5% confidence interval reported. ^‡^*p* value <0.0001, but referred to as nominally significant which denotes *p* < 0.05 but not statistically significant after type I error control or not included in the type I error control strategy.^19^

BDP/FOR/GB, fixed dose combination of beclomethasone, formoterol, and glycopyrronium; FEV1, forced expiratory volume in the first second; FF/UMEC/VI, fixed dose combination of fluticasone furoate, umeclidinium, and vilanterol; FVC, forced volume capacity; HR, hazard ratio; HRQL, health-related quality of life; IC, inspiratory capacity; ICS, inhaled corticosteroids; LABA, long-acting ß_2_ agonist; NS, not significant; OR, odds ratio; RR, risk ratio; SGRQ, St. George’s Respiratory Questionnaire; TDI, transitional dyspnea index.

### FDC triple therapy *versus* open triple therapy

Only two studies evaluated the efficacy of FDC triple therapies *versus* open triple therapies.^14,17^ Both were noninferiority trials. In the study carried out by Bremner and colleagues^17^ the FF/UMEC/VI combination was not inferior to open triple therapy with the same components and, therefore, differences were NS.^17^ Similarly, the TRINITY trial^14^ showed no major differences between both fixed dose and open triple combinations. However, fixed triple dose was associated with similar mean change from baseline in SGRQ total score to open triple at most timepoints, with the exception of weeks 26 and 52, which resulted in a significant difference favoring the open triple therapy in the TRINITY trial.^14^ In addition, the TRINITY trial reported a significant reduction in the rate of moderate-to-severe exacerbations of fixed triple therapy compared with open triple in the subgroup of patients with more than one exacerbation in the previous 12 months with a risk ratio (RR) of 0.71 (CI 95% 0.511–0.995).^14,23^

### Open triple *versus* open triple therapies

Only two recent studies have evaluated two different open triple therapies showing no differences in terms of lung function, health status, rescue medication, daily activities, and exacerbations.^24,25^

### Safety

The summary of the adverse effects recorded in triple therapy FDC clinical trials are summarized in the online supplementary Tables S3 to S5. The proportion of adverse effects was similar between study groups, with COPD worsening being the most common adverse manifestation, the exception being the numerical increase in the number of patients with pneumonia for the ICS containing groups in the IMPACT trial reporting 317 (8%) of cases for FDC triple therapy, 292 (7%) for the LABA/ICS combination and 97 (5%) for the LABA/LAMA combination,^18^ and the intention-to-treat population of FULFIL reporting 19 (2%) for the FDC triple therapy and 7 (<1%) for the LABA/ICS combination.^16^ The TRINITY trial reported 28 (3%) cases of pneumonia for FDC triple therapy *versus* 19 (2%) for tiotropium *versus* 12 (2%) for open triple therapy.^14^ The TRIBUTE trial^15^ reported 28 (4%) cases for FDC triple therapy *versus* 27 (4%) for the LABA/LAMA FDC.

## Discussion

This study analyzes crude efficacy data of triple therapies according to different outcomes and comparators, by performing a comprehensive systematic review with a systematic analysis of the results. In this analysis, we evaluated crude efficacy data in terms of average improvements of the different clinical outcomes assessed. Our data demonstrates consistent improvements for triple combinations *versus* single therapies or double combinations, although with some variability depending on the clinical endpoint considered, the specific combination under evaluation, and the comparison group. Of interest, there were no differences between open and FDC triple therapies or within open triple therapies. Finally, the FDC triple therapies lack of information from a number of clinical outcomes should be explored in the future.

The evaluation of systematic reviews on treatment efficacy helps clinicians because they summarize the evidence and give a global view on the evaluated outcomes. In addition, a meta-analysis with the construct of a mathematical models can help in the understanding of the magnitude of differences in the efficacy outcomes evaluated. Of note, a meta-analysis has its own methodology that is also subjected to potential implications in the evaluation of its results, including the identification and selection of studies, the heterogeneity of results, the availability of information, and the analysis of the data. These caveats in performing and interpreting meta-analyses can yield misleading information.^10^ Alternatively, the summary of crude data directly provided by the clinical trials is a complementary way of presenting pooled data from clinical trials that allows the clinician to better understand the magnitude of the clinical benefits and to evaluate the expected benefits in the patients and finally help in clinical decision making. In the case of triple therapies in COPD, recent meta-analyses have shown efficacy endpoints of fixed triple therapies combining the results in one single analysis.^7–9^ In this analysis we aimed to perform a description of raw data on the efficacy and safety of FDC triple therapies in a systematic way in order to provide clinicians with a joint global view on the efficacy and safety profiles for each combination. In combination, this analysis and the previously published meta-analyses, constitute a thorough analysis of triple therapies in COPD.

There are some methodological considerations to be made to correctly interpret our results. First, the articles evaluated presented a considerable variability in the way the clinical data, including patient characteristics and efficacy outcomes, were presented. Of note, some variables were consistently reported by all of the included trials, while other were not always reported. Specifically, the FDC trials systematically evaluated the blood eosinophils count and previous exacerbations, whereas previous open triple therapies studies did not. Similarly, the evaluation of efficacy parameters was not systematically registered by all trials. In particular, FDC studies did not record peak FEV_1_, FEV_1_ AUC_0-24_, or FEV_1_ 5 min post morning dose. For this analysis, we selected all endpoints frequently reported in previous single or double therapies clinical trials. As the results demonstrate, there are a number of unexplored outcomes, suggesting that there are many aspects still to be explored in FDC triple therapies. Therefore, it is desired that investigators performing clinical trials in COPD reach a consensus on the minimum clinical data that should be included in their analyses, both in terms of the description of included patients and also in the presentation of clinical efficacy.^26^ Finally, we need to consider that a comprehensive evaluation of triple therapies requires an assessment of safety, costs, and device features, that should be performed for a complete analysis. The assessment of the inhaler use is another key aspect of the evaluation. Of interest, a recent study demonstrated that the inadequate management of the inhaler device could be a problem that is underestimated in the real-world practice and could, in turn, be associated with an increased risk of COPD adverse outcomes.^27^ Therefore, patient-centered continuous training and education on inhaler use should be central aspects of patient care in COPD.^28^

A second point to consider is the differences in the methods and the populations analyzed in the included trials of different drug combinations. There were three main differences between the trials of the FDC’s studied that were the follow-up time, the characteristics of the run-in period, and the eligibility criteria. For example, in the FULFIL trial, we used the data at week 26, although a cohort of 430 patients completed the 52 weeks follow-up.^16^ In addition, both of the analyzed FDC’s presented similar criteria for eligible patients but with some differences, resulting in patients with different severity. Finally, the inclusion of a run-in period is common in clinical trials because it allows ineligible or noncompliant participants to be screened out, ensuring that participants are in a stable condition, and providing baseline observations under the same conditions.^29^ Although FDC trials required a 2 week run-in period, the FF/UMEC/VI studies did not modify the previous medications during the run-in and there were cases previously using triple therapy (38% in the IMPACT trial). This aspect in combination with a considerable proportion of ICS users pre-trial has been suggested as a relevant methodological consideration in the IMPACT trial.^30^ Although this might impact on the results, whether this effect after discontinuing ICS is transient as reported,^31^ or prolonged over time, has not been sufficiently explored. Therefore, the evaluation of this effect on the long term during the trial is still needs to be evaluated. As a result, a raw direct comparison between studies appears unfeasible and was avoided in our study.

Third, another methodological aspect that is worth highlighting is that these trials excluded patients with a current but not past diagnosis of asthma. Therefore, some patients with a past diagnosis of asthma could have been included in all trials and this could affect the results in favor of ICS containing regimens. Unfortunately, none of the FDC triple therapy trials reported the distribution of patients with a previous diagnosis of asthma between the different treatment arms.

The assessment of mean values as outcome measures requires discussion. Although it is accepted that the mean improvement is a simple way to show the overall pharmacological response, mean values represent a simplification of a more complex reality. In daily practice, it is more interesting to be able to evaluate the variability of this response rather than the average improvement. In addition, it has been shown that due to the rigid inclusion and exclusion criteria, the population in a clinical trial may not represent the clinical reality of a disease in a real-world setting.^32^ Recently, different trials have highlighted this different response at the patient level when evaluating double bronchodilation drug combinations.^33,34^ Therefore, complementary methods for showing improvement in clinical trials as the number of patients that reached the MCID results is necessary.

The importance of the impact of FDC triple therapies on exacerbations requires discussion. The evaluation of these combination on all exacerbations was only reported by the FULFIL trial,^16^ the rest of the trials focusing on moderate-severe exacerbations only. Globally considered, these trials reported a reduction ranging from 15% to 35% for all comparisons.^16,18^ Of note, the evaluation of FDC triple *versus* a LAMA was only reported in the TRINITY trial^14^ with a RR of 0.80 (CI 95% 0.69–0.92) for the annualized rate of exacerbations and 0.84 (CI 95% 0.72–0.97) for the time to the first exacerbation. This trial evaluated the potential role of adding a LABA/ICS to patients receiving a LAMA. Of interest, the opposite situation evaluating the addition of a LAMA to ICS/LABA was explored in three FDC trials showing similar figures albeit with some variability.^13,16,18^ These results are interesting because we know from previous trials that a LAMA is similar to a LABA/ICS in the prevention of exacerbations.^35^ Therefore, a similar impact might be expected when combining them together one way or the other. However, the addition of an ICS to a LABA/LAMA combination resulted in a reduction of 25% in the rate of exacerbations for FF/UMEC/VI and 16% for BDP/FOR/GB. The time to the first exacerbation was also different between the trials reporting a HR of 0.84 (CI 95% 0.78−0.91) but with no statistical association in the TRIBUTE trial.^15^

One aspect of controversy in FDC trials is the lack of a consistent relationship with eosinophil blood count. Our analysis did not include the results according to the basal eosinophil count as previous reports have done.^8^ Of interest, blood eosinophil count was not relevant in these analyses when adding an ICS, challenging the strategies in current recommendation documents.^36^ Although previous evidence is consistent, showing a better response to ICS with increased blood eosinophils, we must bear in mind that all of this evidence comes from *post-hoc*, secondary prespecified, and data modeling analyses.^37^ Therefore, to the best of our knowledge, at present there is insufficient evidence to recommend that blood eosinophils should be used to predict future exacerbation risk on an individual basis in COPD patients.^36^

Other interesting subanalysis results were those reported in the IMPACT trial that showed a significant difference in the reduction in the rate of exacerbation regardless of the patient’s smoking status.^18^ Of interest, BDP/FOR/GB trials presented a significant increase in the prevention of exacerbations in ex-smokers when compared with LABA/ICS^13^ and tiotropium,^14^ but not with the LABA/LAMA combination.^15^ Although studies on the use of ICS in asthma have shown a short-term improvement in lung function and a reduction in anti-inflammatory effects in active smokers compared with non-smokers,^38,39^ the association in COPD is less studied. Recently, an effect similar to the effects observed in patients with asthma has been described and, therefore, affects the achievement of important clinical outcomes in patients with COPD.^40^

Another interesting subanalysis is by clinical phenotype. In the TRIBUTE trial^15^ patients with chronic bronchitis who received BDP/FOR/GB had a significantly reduced exacerbation rate compared with LABA/LAMA and the adjusted rate ratios were NS in patients with emphysema and in those with mixed bronchitis and emphysema. However, the assignment of patients to chronic bronchitis or emphysema groups was based on the opinion of the investigator, without being supported by imaging or lung function testing. Therefore, these results must be viewed with caution and should be confirmed in future studies.

A more relevant unexpected result reported was the decrease in all-cause mortality for FF/UMEC/VI when compared with UMEC/VI in the IMPACT trial.^18^ This should be viewed with caution. Although the potential impact on mortality of a triple therapy has previously been reported,^41^ in the IMPACT trial this mortality analysis was reported as an exploratory analysis, and was not included in the primary or secondary objectives of the trial, for the prespecified on treatment population, with no adjustment for multiplicity and with an unadjusted *p* value of 0.01. Of interest, a recent pooled analysis of the BDP/FOR/GB triple combination therapy showed this effect only for nonrespiratory cause of mortality.^42^ Of note, there are a number of well-known factors that are associated with mortality in COPD patients.^43–45^ Therefore, additional studies are needed to explore the impact of triple therapy on mortality as a primary outcome.

## Conclusion

The current study is a systematic review summarizing all of the available clinical trials that focus on the efficacy of open and FDC triple therapies in patients with COPD. Average changes reported here highlight consistent improvements with the use of fixed triple therapy when compared with other single or double therapies in a specific population of severe COPD patients, with no greater differences with open triple therapies combinations. These results will help physicians improve their understanding of the magnitude of the clinical benefits they may expect for help in making clinical decisions, that should be patient-centered.

## Supplemental Material

Author_Response_1 – Supplemental material for Triple therapy for COPD: a crude analysis from a systematic review of the evidence Click here for additional data file. Supplemental material, Author_Response_1 for Triple therapy for COPD: a crude analysis from a systematic review of the evidence by Jose Luis Lopez-Campos, Laura Carrasco-Hernandez, Esther Quintana-Gallego, Carmen Calero-Acuña, Eduardo Márquez-Martín, Francisco Ortega-Ruiz and Joan B. Soriano in Therapeutic Advances in Respiratory Disease

Online_supplement – Supplemental material for Triple therapy for COPD: a crude analysis from a systematic review of the evidence Click here for additional data file. Supplemental material, Online_supplement for Triple therapy for COPD: a crude analysis from a systematic review of the evidence by Jose Luis Lopez-Campos, Laura Carrasco-Hernandez, Esther Quintana-Gallego, Carmen Calero-Acuña, Eduardo Márquez-Martín, Francisco Ortega-Ruiz and Joan B. Soriano in Therapeutic Advances in Respiratory Disease

Reviewer_1_v.1 – Supplemental material for Triple therapy for COPD: a crude analysis from a systematic review of the evidence Click here for additional data file. Supplemental material, Reviewer_1_v.1 for Triple therapy for COPD: a crude analysis from a systematic review of the evidence by Jose Luis Lopez-Campos, Laura Carrasco-Hernandez, Esther Quintana-Gallego, Carmen Calero-Acuña, Eduardo Márquez-Martín, Francisco Ortega-Ruiz and Joan B. Soriano in Therapeutic Advances in Respiratory Disease

Reviewer_1_v.2 – Supplemental material for Triple therapy for COPD: a crude analysis from a systematic review of the evidence Click here for additional data file. Supplemental material, Reviewer_1_v.2 for Triple therapy for COPD: a crude analysis from a systematic review of the evidence by Jose Luis Lopez-Campos, Laura Carrasco-Hernandez, Esther Quintana-Gallego, Carmen Calero-Acuña, Eduardo Márquez-Martín, Francisco Ortega-Ruiz and Joan B. Soriano in Therapeutic Advances in Respiratory Disease

Reviewer_2_v.1 – Supplemental material for Triple therapy for COPD: a crude analysis from a systematic review of the evidence Click here for additional data file. Supplemental material, Reviewer_2_v.1 for Triple therapy for COPD: a crude analysis from a systematic review of the evidence by Jose Luis Lopez-Campos, Laura Carrasco-Hernandez, Esther Quintana-Gallego, Carmen Calero-Acuña, Eduardo Márquez-Martín, Francisco Ortega-Ruiz and Joan B. Soriano in Therapeutic Advances in Respiratory Disease

Reviewer_2_v.2 – Supplemental material for Triple therapy for COPD: a crude analysis from a systematic review of the evidence Click here for additional data file. Supplemental material, Reviewer_2_v.2 for Triple therapy for COPD: a crude analysis from a systematic review of the evidence by Jose Luis Lopez-Campos, Laura Carrasco-Hernandez, Esther Quintana-Gallego, Carmen Calero-Acuña, Eduardo Márquez-Martín, Francisco Ortega-Ruiz and Joan B. Soriano in Therapeutic Advances in Respiratory Disease
