# Supplementary material for: Triple therapy for COPD: a crude analysis from a systematic review of the evidence
Source: Ther Adv Respir Dis. 2019 Nov 6;13:1753466619885522. doi: 10.1177/1753466619885522 (PMC7000908; doi:10.1177/1753466619885522)
Supplement: Online_supplement – Supplemental material for Triple therapy for COPD: a crude analysis from a systematic review of the evidence [file Online_supplement.pdf]

Table S1. Summary of the design of the trials with open triple combinations

Studies	Design	Treatment arms	Inhalers	Duration	Primary outcome	N *
Multiple comparators						
Hoshino M, Ohtawa J. Respiration 2013 [1]	RCT	FP/SAL + TIO<br>FP/SAL<br>SAL<br>TIO	Accuhaler + Handihaler<br>Accuhaler<br>Accuhaler<br>Handihaler	16 weeks	Airway dimensions by CT scan	68
Aaron SD, et al. Ann Intern Med 2007 OPTIMAL [2]	RCT	FP/SAL + TIO<br>SAL + TIO<br>TIO	pMDI + Handihaler<br>pMDI + Handihaler<br>Handihaler	1 year	% patients with an exacerbation	449
Saito T, et al. Int J COPD 2015 EAGLE [3]	Crossover	FP/SAL + TIO<br>FP/SAL<br>TIO	Accuhaler + Handihaler<br>Accuhaler<br>Handihaler	4 weeks	airway conductance AUC_{0-4}	53
Singh D, et al. Thorax 2008 [4]	Crossover	FP/SAL + TIO<br>FP/SAL<br>TIO	Accuhaler + Handihaler<br>Accuhaler<br>Handihaler	2 weeks	post-dose specific airways conductance AUC_{0-4}	30

Studies	Design	Treatment arms	Inhalers	Duration	Primary outcome	N *
Cazzola M, et al. Respir Med 2007 [5]	RCT	FP/SAL + TIO<br>FP/SAL<br>TIO	Accuhaler + Handihaler<br>Accuhaler<br>Handihaler	3 months	Trough FEV_1	90
Triple vs LAMA						
Hoshino M, Ohtawa J. Respirology 2011 [6]	RCT	FP/SAL + TIO<br>TIO	Accuhaler + Handihaler<br>Handihaler	142 weeks	Airway dimensions by CT scan	30
Maltais F, et al. Eur Respir J 2013 [7]	RCT	FP/SAL + TIO<br>TIO	Accuhaler + Handihaler<br>Handihaler	8 weeks	Exercise endurance time	255
Hanania N, et al. Respir Med 2012 [8]	RCT	FP/SAL + TIO<br>TIO	Accuhaler + Handihaler<br>Handihaler	24 weeks	Trough FEV_1	342
Jung KS, et al. Respir Med 2012 [9]	RCT	FP/SAL + TIO<br>TIO	Accuhaler + Handihaler<br>Handihaler	24 weeks	Trough FEV_1	479
Feng JF, et al. Medicine 2018 [10]	RCT	BUD/FORM + TIO<br>TIO	Turbuhaler + handihaler<br>Handihaler	12 weeks	Dyspnea on the 6-minute walk test	113

Studies	Design	Treatment arms	Inhalers	Duration	Primary outcome	N *
Lee SD, et al. Respirology 2015 [11]	RCT	BUD/FORM + TIO<br>TIO	Turbuhaler + handihaler<br>Handihaler	12 weeks	Trough FEV_1	578
Williamson PA, et al. Chest 2010 [12]	Crossover	BUD/FORM + TIO<br>TIO	Turbuhaler + handihaler<br>Handihaler	2 weeks	Trough FEV_1	22
Welte T, et al. AJRCCM 2009<br>CLIMB [13]	RCT	BUD/FOR + TIO<br>TIO	Turbuhaler + handihaler<br>Handihaler	12 weeks	Trough FEV_1	660
Triple vs LABA/ICS						
Frith PA, et al. Thorax 2015<br>GLISTEN [14]	RCT	FP/SAL + TIO<br>FP/SAL + GB<br>FP/SAL	Accuhaler + Handihaler<br>Accuhaler + Breezhaler<br>Accuhaler	12 weeks	Trough FEV_1	773
Siler TM, et al. COPD 2016 [15]	RCT	FP/SAL + UMEC<br>FP/SAL	Accuhaler + Ellipta<br>Accuhaler	12 weeks	Trough FEV_1	1225
Singh D, et al. Respir Med 2016<br>TRIDENT [16]	Crossover	BDP/FOR + GB<br>BDP/FOR	pMDI + pMDI<br>pMDI	7 days	FEV_1 $AUC_{0\text{-}12h}$	178

Studies	Design	Treatment arms	Inhalers	Duration	Primary outcome	N *
Siler TM, et al. Respir Med 2015 [17]	RCT	FF/VI + UMEC<br>FF/VI	Ellipta + Ellipta<br>Ellipta	12 weeks	Trough FEV_1	1238

* N: referred to the number of subjects randomized to any of the treatment arms for RCT and referred to the complete population randomized for the crossover studies. RCT: Randomized controlled trial. BDP: beclomethasone dipropionate; FOR: formoterol fumarate; GB: glycopyrronium bromide; IND: indacaterol; FF: Fluticasone furoate; UMEC: umeclidinium; VI: vilanterol; BUD: budesonide; FOR: formoterol; TIO: tiotropium; SAL: salmeterol; FP: fluticasone propionate; FEV_1: forced expiratory volume in the first second; SGRQ: St. George's Respiratory Questionnaire;: pMDI: pressured metered dose inhaler; AUC: Area under the curve; CT: computed tomography

Table S2. Summary of characteristics of the patients of the trials with open triple combinations

Study	Age (years)	Male (%)	Smokers (%)	ICS (%)	FEV_1 (L)	FEV_1 (%)	Reversibility (%)	Exac./yr	≥ 2 exac. (%)
Multiple comparators									
Hoshino M, Ohtawa J. Respiration 2013 [1]	73	86.6	–	–	1.38	–	–	–	–
Aaron SD, et al. Ann Intern Med 2007 OPTIMAL [2]	67.5	57.9	32.4	72.8	1.12	42.2	–	– *	– *
Saito T, et al. Int J COPD 2015 EAGLE [3]	67.3	98	36	–	–	59.3	4.1	0.1	–
Singh D, et al. Thorax 2008 [4]	62.7	77.0	47.0	53.3		47.1	6.78	0.2	–
Cazzola M, et al. Respir Med 2007 [5]	66.9	86.6	80.0	66.6	–	39.0	12.8	–	–
Triple vs LAMA									
Hoshino M, Ohtawa J. Respirology 2011 [6]	73	87.5	–	–	1.36	64.6	–	–	–
Maltais F, et al. Eur Respir J 2013 [7]	–	–	–	–	–	–	–	–	–
Hanania N, et al. Respir Med 2012 [8]	61.3	50.0	59.0	–	1.67	–	–	–	6.3
Jung KS, et al. Respir Med 2012 [9]	67.0	97.3	–	–	1.22	47.4	–	–	–

Study	Age (years)	Male (%)	Smokers (%)	ICS (%)	FEV_1 (L)	FEV_1 (%)	Reversibility (%)	Exac./yr	≥ 2 exac. (%)
Feng JF, et al. Medicine 2018 [10]	64.9	77.8	54.0	52.8	–	45.1	–	–	–
Lee SD, et al. Respirology 2015 [11]	66.6	97.2	–	–	–	35.8	–	–	–
Williamson PA, et al. Chest 2010 [12]	65.0	63.1	–	–	–	42.0	–	–	–
Welte T, et al. AJRCCM 2009; CLIMB [13]	62.4	76.3	41.6	66.5	1.1	38.1	5.9	1.4	–
Triple vs LABA/ICS									
Frith PA, et al. Thorax 2015; GLISTEN [14] **	68.0	62.0	35.7	66.3	1.55	57.35	22.41	–	35.7 (> 1)
Siler TM, et al. COPD 2016 [15] Study 1	62.7	65.0	50.0	55.0	1.31	46.8	16.2	–	–
Siler TM, et al. COPD 2016 [15] Study 2	64.5	69.0	36.0	59.0	1.15	43.9	16.1	–	–
Singh D, et al. Respir Med 2016; TRIDENT [16]	62.7	66.9	54.5	–	1.41	48.9	16.4	–	–
Siler TM, et al. Respir Med 2015 [17] Study 1	64.9	67.4	39.3	–	–	44.2	14.8	–	–
Siler TM, et al. Respir Med 2015 [17] Study 12	62.6	65.5	58.2	–	–	46.3	13.2	–	–

Results referred to the triple therapy arm in randomized controlled trials and for the complete population in crossover studies.

* At least 1 exacerbation of COPD that required treatment with systemic steroids or antibiotics within the 12 months before randomization, as per protocol

** Results referred to the salmeterol/fluticasone + tiotropium combination.

Table S3. Description of the main adverse effects with BDP/FF/GB fixed-dose triple therapy expressed as percentages

	TRILOGY[18]	TRINITY [19]	TRIBUTE [20]
**Treatment-emergent adverse events**	**54**	**55**	**64**
COPD	31	33	36
Nasopharyngitis	6	5	6
Pneumonia	3	3	4
Hypertension	3		2
Headache	2	4	6
Ischaemic heart disease	1	2	
Viral respiratory tract infection	2	1	3
Oral candidiasis	2		
Cough		2	2
Dyspnoea			3
Back pain			3
Cardiac failure			2
Ischaemic heart disease			1
**Serious treatment-emergent adverse events**	**15**	**13**	**15**
COPD	10	7	8
Pneumonia	2	2	2
Ischaemic heart disease	<1	1	<1
Cardiac failure	1	<1	1
Death			<1
Atrial fibrillation			0
Respiratory failure			<1

Lung neoplasm			1
**Treatment-related adverse events**	**4**	**2**	**6**
Oral candidiasis	1	<1	2
Muscle spasms	1	1	
Dry mouth	1	<1	<1
Dysphonia		<1	
Cough			<1
**Serious treatment-related adverse events**	**<1**	**0**	**<1**
**Severe treatment-related adverse events**	**11**	**8**	**11**
**Treatment-emergent adverse events leading to study drug discontinuation**	**5**	**3**	**5**
**Treatment-emergent adverse events leading to death**	**2**	**2**	**2**
**MACE (Major adverse cardiovascular events)**	**2**	**2**	

* MACE: Major adverse cardiovascular events, includes acute myocardial infarction, arrhythmias, cardiovascular death, heart failure, and stroke. Treatment emergent adverse effects were those captured throughout the study. Treatment-related adverse effects were all events judged by the investigator as having reasonable causal association to a medical product.

Table S4. Description of the main adverse effects with FF/UMEC/VI fixed-dose triple therapy expressed as percentages.

	FULFIL [21]	IMPACT [22]
Adverse events of special interest*		
Cardiovascular effects	4.3	11
Pneumonia	2.2	8
Local steroid effects	2.1	
Anticholinergic syndrome	1.8	4
Hypersensitivity	1.1	
Hyperglycemia/diabetes	0.5	
Decreased bone mineral density	0.4	
lower respiratory tract infection (excluding pneumonia)	0.3	5
Ocular effects	0.1	
Urinary retention	0.1	
Asthma/bronchospasm	0	<1
Cardiovascular effects	11	
Urinary retention		<1

* Adverse events of special interest are based on an analysis of a group of prespecified adverse events that are associated with the use of inhaled glucocorticoids, long-acting muscarinic antagonists, or long-acting β_2-agonists

Table S5. Description of the main adverse effects with BUD/FOR/GB fixed-dose triple therapy expressed as percentages.

	KRONOS [23]
Nasopharyngitis	8
Upper respiratory tract infection	10
Chronic obstructive pulmonary disease	3
Bronchitis	3
Muscle spasms	3
Dysphonia	3
Hypertension	2
Dyspnea	1
Back pain	1
Nausea	1

References

1. Hoshino M and Ohtawa J. Effects of tiotropium and salmeterol/fluticasone propionate on airway wall thickness in chronic obstructive pulmonary disease. *Respiration; international review of thoracic diseases* 2013; 86: 280-287. DOI: 10.1159/000351116.
2. Aaron SD, Vandemheen KL, Fergusson D, et al. Tiotropium in combination with placebo, salmeterol, or fluticasone-salmeterol for treatment of chronic obstructive pulmonary disease: a randomized trial. *Annals of internal medicine* 2007; 146: 545-555. DOI: 10.7326/0003-4819-146-8-200704170-00152.
3. Saito T, Takeda A, Hashimoto K, et al. Triple therapy with salmeterol/fluticasone propionate 50/250 plus tiotropium bromide improve lung function versus individual treatments in moderate-to-severe Japanese COPD patients: a randomized controlled trial - Evaluation of Airway sGaw after treatment with tripLE. *International journal of chronic obstructive pulmonary disease* 2015; 10: 2393-2404. DOI: 10.2147/COPD.S89948.
4. Singh D, Brooks J, Hagan G, et al. Superiority of "triple" therapy with salmeterol/fluticasone propionate and tiotropium bromide versus individual components in moderate to severe COPD. *Thorax* 2008; 63: 592-598. 2008/02/05. DOI: 10.1136/thx.2007.087213.
5. Cazzola M, Ando F, Santus P, et al. A pilot study to assess the effects of combining fluticasone propionate/salmeterol and tiotropium on the airflow obstruction of patients with severe-to-very severe COPD. *Pulmonary pharmacology & therapeutics* 2007; 20: 556-561. DOI: 10.1016/j.pupt.2006.06.001.
6. Hoshino M and Ohtawa J. Effects of adding salmeterol/fluticasone propionate to tiotropium on airway dimensions in patients with chronic obstructive pulmonary disease. *Respirology* 2011; 16: 95-101. DOI: 10.1111/j.1440-1843.2010.01869.x.
7. Maltais F, Mahler DA, Pepin V, et al. Effect of fluticasone propionate/salmeterol plus tiotropium versus tiotropium on walking endurance in COPD. *The European respiratory journal : official journal of the European Society for Clinical Respiratory Physiology* 2013; 42: 539-541. DOI: 10.1183/09031936.00074113.
8. Hanania NA, Crater GD, Morris AN, et al. Benefits of adding fluticasone propionate/salmeterol to tiotropium in moderate to severe COPD. *Respiratory medicine* 2012; 106: 91-101. DOI: 10.1016/j.rmed.2011.09.002.
9. Jung KS, Park HY, Park SY, et al. Comparison of tiotropium plus fluticasone propionate/salmeterol with tiotropium in COPD: a randomized controlled study. *Respiratory medicine* 2012; 106: 382-389. DOI: 10.1016/j.rmed.2011.09.004.
10. Feng JF, Ding GR, Xie YZ, et al. Efficacy of budesonide/formoterol and tiotropium combination for the treatment of Chinese patients with chronic obstructive pulmonary disease. *Medicine (Baltimore)* 2018; 97: e10841. DOI: 10.1097/MD.0000000000010841.
11. Lee SD, Xie CM, Yunus F, et al. Efficacy and tolerability of budesonide/formoterol added to tiotropium compared with tiotropium alone in patients with severe or very severe COPD: A randomized, multicentre study in East Asia. *Respirology* 2016; 21: 119-127. DOI: 10.1111/resp.12646.
12. Williamson PA, Short PM, Clearie KL, et al. Paradoxical trough effects of triple therapy with budesonide/formoterol and tiotropium bromide on pulmonary function outcomes in COPD. *Chest* 2010; 138: 595-604. DOI: 10.1378/chest.10-0247.
13. Welte T, Miravitlles M, Hernandez P, et al. Efficacy and tolerability of budesonide/formoterol added to tiotropium in patients with chronic obstructive pulmonary disease. *American journal of respiratory and critical care medicine* 2009; 180: 741-750. DOI: 10.1164/rccm.200904-0492OC.
14. Frith PA, Thompson PJ, Ratnavadivel R, et al. Glycopyrronium once-daily significantly improves lung function and health status when combined with salmeterol/fluticasone in patients with COPD: the GLISTEN study, a randomised

controlled trial. *Thorax* 2015; 70: 519-527. 2015/04/05. DOI: 10.1136/thoraxjnl-2014-206670.
15. Siler TM, Kerwin E, Singletary K, et al. Efficacy and Safety of Umeclidinium Added to Fluticasone Propionate/Salmeterol in Patients with COPD: Results of Two Randomized, Double-Blind Studies. *Copd* 2016; 13: 1-10. DOI: 10.3109/15412555.2015.1034256.
16. Singh D, Schroder-Babo W, Cohuet G, et al. The bronchodilator effects of extrafine glycopyrronium added to combination treatment with beclometasone dipropionate plus formoterol in COPD: A randomised crossover study (the TRIDENT study). *Respiratory medicine* 2016; 114: 84-90. DOI: 10.1016/j.rmed.2016.03.018.
17. Siler TM, Kerwin E, Sousa AR, et al. Efficacy and safety of umeclidinium added to fluticasone furoate/vilanterol in chronic obstructive pulmonary disease: Results of two randomized studies. *Respiratory medicine* 2015; 109: 1155-1163. DOI: 10.1016/j.rmed.2015.06.006.
18. Singh D, Papi A, Corradi M, et al. Single inhaler triple therapy versus inhaled corticosteroid plus long-acting beta2-agonist therapy for chronic obstructive pulmonary disease (TRILOGY): a double-blind, parallel group, randomised controlled trial. *Lancet* 2016; 388: 963-973. DOI: 10.1016/S0140-6736(16)31354-X.
19. Vestbo J, Papi A, Corradi M, et al. Single inhaler extrafine triple therapy versus long-acting muscarinic antagonist therapy for chronic obstructive pulmonary disease (TRINITY): a double-blind, parallel group, randomised controlled trial. *Lancet* 2017; 389: 1919-1929. DOI: 10.1016/S0140-6736(17)30188-5.
20. Papi A, Vestbo J, Fabbri L, et al. Extrafine inhaled triple therapy versus dual bronchodilator therapy in chronic obstructive pulmonary disease (TRIBUTE): a double-blind, parallel group, randomised controlled trial. *Lancet* 2018; 391: 1076-1084. DOI: 10.1016/S0140-6736(18)30206-X.
21. Lipson DA, Barnacle H, Birk R, et al. FULFIL Trial: Once-Daily Triple Therapy for Patients with Chronic Obstructive Pulmonary Disease. *American journal of respiratory and critical care medicine* 2017; 196: 438-446. DOI: 10.1164/rccm.201703-0449OC.
22. Lipson DA, Barnhart F, Brealey N, et al. Once-Daily Single-Inhaler Triple versus Dual Therapy in Patients with COPD. *The New England journal of medicine* 2018; 378: 1671-1680. DOI: 10.1056/NEJMoa1713901.
23. Ferguson GT, Rabe KF, Martinez FJ, et al. Triple therapy with budesonide/glycopyrrolate/formoterol fumarate with co-suspension delivery technology versus dual therapies in chronic obstructive pulmonary disease (KRONOS): a double-blind, parallel-group, multicentre, phase 3 randomised controlled trial. *The lancet Respiratory medicine* 2018; 6: 747-758. 2018/09/21. DOI: 10.1016/s2213-2600(18)30327-8.
