# Supplementary material for: Triple therapy for COPD: a crude analysis from a systematic review of the evidence
Source: Ther Adv Respir Dis. 2019 Nov 6;13:1753466619885522. doi: 10.1177/1753466619885522 (PMC7000908; doi:10.1177/1753466619885522)
Supplement: Reviewer_1_v.1 – Supplemental material for Triple therapy for COPD: a crude analysis from a systematic review of the evidence [file Reviewer_1_v.1.pdf]

Reviewer 1 v.1

Comments to the Author

This study seems to be well performed and presented well.

But there are several points to be addressed.

The major issues

-The authors presented key studies on efficacy of FDC and open triple therapies, but, unfortunately, one important and relevant study – KRONOS – was not mentioned. The inclusion of this study into analysis is desirable.

The minor issues

-The authors discussed the role of blood eosinophil counts as predictor of the response to ICS, but also smoking status (IMPACT) and bronchitis phenotype (TRIBUTE) could be important modifiers of treatment response to ICS. The issues should be discussed.

-There are some blank spaces in table 2 (Rescue medication, Time to first exacerbation, etc.) and in table 3 (Time to first exacerbation)
