# Supplementary material for: Triple therapy for COPD: a crude analysis from a systematic review of the evidence
Source: Ther Adv Respir Dis. 2019 Nov 6;13:1753466619885522. doi: 10.1177/1753466619885522 (PMC7000908; doi:10.1177/1753466619885522)
Supplement: Reviewer_1_v.2 – Supplemental material for Triple therapy for COPD: a crude analysis from a systematic review of the evidence [file Reviewer_1_v.2.pdf]

Reviewer 1 v.2

Comments to the Author

All reviewer's comments were answered, so I recommend this paper for the publication.
