# Supplementary material for: Triple therapy for COPD: a crude analysis from a systematic review of the evidence
Source: Ther Adv Respir Dis. 2019 Nov 6;13:1753466619885522. doi: 10.1177/1753466619885522 (PMC7000908; doi:10.1177/1753466619885522)
Supplement: Reviewer_2_v.1 – Supplemental material for Triple therapy for COPD: a crude analysis from a systematic review of the evidence [file Reviewer_2_v.1.pdf]

Reviewer 2 v.2

Comments to the Author

Authors provide a clinical summary review focused on DBPC trials for triple therapy (closed or open) for COPD. They focused on three studies with fixed dose BDP/FF/GB (TRILOGY, TRINITY, TRIBUTE) and three studies with fixed dose FF/UMEC/VI (FULFIL, Bremmer et. al and IMPACT). While they also briefly summarize several open triple studies (listed in Table S1), none of the open triple studies were long term or able to report exacerbation outcomes.

Therefore, the authors discussion primarily relates to the six larger and longer studies listed above.

Unfortunately, some of the most recently published studies on triple therapy for COPD, including the KRONOS study (published in about SEP2018) and the recent ETHOS study (press release in August 2019) on BUD/GP/FF were not included. These studies helped confirm a widening role for triple therapies for COPD, extending across current GOLD B and GOLD D groups, and with blood eosinophils levels of 150 and above (potentially even lower). Therefore, it is unfortunate that authors are publishing with a one-year delay (in late 2019) a review with a MS search date a year prior (1AUG 2018).

Nevertheless, I do recommend this MS for publication in TARD. The authors correctly identify important articles published prior to 1AUG2018 related to triple therapies for COPD. Their summary correctly confirms that triples have been shown to benefit trough FEV1, other airflow measures, TDI, SGRQ responders, as well as reducing the rate of moderate to severe COPD exacerbations. Reduction in all-cause mortality (in IMPACT) are also intriguing, and are described in the discussion.

Suggestions:

The safety tables presented in Table S3 and S4 present only rates (%) of reported AEs for the triple therapy study arm, without presenting the similar AE rates for the relevant control group. Control group AE rates should be added to give readers a perspective on the AE rates.

Thank you for giving me the opportunity to review this MS. I do recommend it for consideration for publication in TARD with only minor changes needed.

Reviewer #2
