# Supplementary material for: Triple therapy for COPD: a crude analysis from a systematic review of the evidence
Source: Ther Adv Respir Dis. 2019 Nov 6;13:1753466619885522. doi: 10.1177/1753466619885522 (PMC7000908; doi:10.1177/1753466619885522)
Supplement: Reviewer_2_v.2 – Supplemental material for Triple therapy for COPD: a crude analysis from a systematic review of the evidence [file Reviewer_2_v.2.pdf]

Reviewer 2 v.2

Comments to the Author

Authors conducted an updated literature search on 07SEP2019, and found the more recently published KRONOS study on BUD/GP/FF, to add these data into their clinical summaries.

I find these changes are helpful.

I can now recommend this MS for publication in Therapeutic Advance in Respiratory Disease.

Thanks.

Reviewer #2
