# Supplementary material for: Triple therapy for COPD: a crude analysis from a systematic review of the evidence
Source: Ther Adv Respir Dis. 2019 Nov 6;13:1753466619885522. doi: 10.1177/1753466619885522 (PMC7000908; doi:10.1177/1753466619885522)
Supplement: Author_Response_1 – Supplemental material for Triple therapy for COPD: a crude analysis from a systematic review of the evidence [file Author_Response_1.pdf]

Author Response 1

Reviewer: 1

Comments to the Author

This study seems to be well performed and presented well. But there are several points to be addressed.

The major issues

COMMENT: The authors presented key studies on efficacy of FDC and open triple therapies, but, unfortunately, one important and relevant study – KRONOS – was not mentioned. The inclusion of this study into analysis is desirable.

RESPONSE: We would like to thank the reviewer for the evaluation. The reviewer is correct. At that time, the problem was that the systematic search was done on August 3rd, 2018, and the KRONOS Study was published online on September 16th, 2018. We agree with the reviewer that is necessary to include it in a systematic review. To be honest, after KRONOS publication date, we run the search again, but we initially decided not to include KRONOS for several reasons. KRONOS is a complex trial. Primary and secondary endpoints and treatment comparisons of interest differed according to regulatory registration requirements between Europe/Canada and USA. As a consequence, KRONOS study reported the majority of results over 24 weeks instead of at 24 weeks, as in the rest of the trials. Since we aimed to report comparison between treatment arms at the end of the trial, many results were not comparable and accordingly were not to be included in the tables. Additionally, a strong control of the type I error rate was maintained in the analysis of the KRONOS study. As a consequence, a difference was termed as nominally significant when $p<0.05$ but not statistically significant after type I error control or not included in the type I error control strategy, which is very confusing. We understand, however, that a systematic review should include it. We have now done so. We have updated the systematic search for that combination and updated the whole manuscript accordingly, with several notes explaining these peculiarities.

COMMENT: The minor issues

-The authors discussed the role of blood eosinophil counts as predictor of the response to ICS, but also smoking status (IMPACT) and bronchitis phenotype (TRIBUTE) could be important modifiers of treatment response to ICS. The issues should be discussed.

RESPONSE: We agree with the reviewer. We have now included a comment on these two relevant topics.

COMMENT: -There are some blank spaces in table 2 (Rescue medication, Time to first exacerbation, etc.) and in table 3 (Time to first exacerbation)

RESPONSE: Thanks for noticing this. We have now corrected it.

Reviewer: 2

COMMENT: Authors provide a clinical summary review focused on DBPC trials for triple therapy (closed or open) for COPD. They focused on three studies with fixed dose BDP/FF/GB (TRILOGY, TRINITY, TRIBUTE) and three studies with fixed dose FF/UMEC/VI (FULFIL, Bremmer et. al and IMPACT). While they also briefly summarize several open triple studies (listed in Table S1), none of the open triple studies were long term or able to report exacerbation outcomes. Therefore, the authors discussion primarily relates to the six larger and longer studies listed above. Unfortunately, some of the most recently published studies on triple therapy for COPD, including the KRONOS study (published in about SEP2018) and the recent ETHOS study (press release in August 2019) on BUD/GP/FF were not included. These studies helped confirm a widening role for triple therapies for COPD, extending across current GOLD B and GOLD D groups, and with blood eosinophils levels of 150 and above (potentially even lower). Therefore, it is unfortunate that authors are publishing with a one-year delay (in late 2019) a review with a MS search date a year prior (1AUG 2018). Nevertheless, I do recommend this MS for publication in TARD. The authors correctly identify important articles published prior to 1AUG2018 related to triple therapies for COPD. Their summary correctly confirms that triples have been shown to benefit trough FEV1, other airflow measures, TDI, SGRQ responders, as well as reducing the rate of moderate to severe COPD exacerbations. Reduction in all-cause mortality (in IMPACT) are also intriguing, and are described in the discussion.

ANSWER: We would like to thank the reviewer for evaluating the manuscript and the positive comments. As responded to the previous reviewer, initially the problem was that the systematic search was done on August 3rd, 2018, and the KRONOS Study was published online on September 16th, 2018. We agree with the reviewer that is necessary to include it in a systematic review. To be honest, after KRONOS publication date, we run the search again, but we initially decided not to include KRONOS for several reasons. KRONOS is a complex trial. Primary and secondary endpoints and treatment comparisons of interest differed according to regulatory registration requirements between Europe/Canada and USA. As a consequence, KRONOS study reported the majority of results over 24 weeks instead of at 24 weeks, as in the rest of the trials. Since we aimed to report comparison between treatment arms at the end of the trial, many results were not comparable and accordingly were not to be included in the tables. Additionally, a strong control of the type I error rate was maintained in the analysis of the KRONOS study. As a consequence, a difference was termed as nominally significant when $p<0.05$ but not statistically significant after type I error control or not included in the type I error control strategy, which is very confusing. We understand, however, that a systematic review should include it. We have now done so. We have updated the systematic search for that combination and updated the whole manuscript accordingly, with several notes explaining these peculiarities. Unfortunately, the mentioned ETHOS trial has not appeared in any of the searches.

COMMENT: Suggestions: The safety tables presented in Table S3 and S4 present only rates (%) of reported AEs for the triple therapy study arm, without presenting the similar AE rates for the relevant control group. Control group AE rates should be added to give readers a perspective on the AE rates.

RESPONSE: Yes, we did this on purpose. The aim of these tables was to show specifically AE rates with triple to give access in not complex tables the relevant information. The readers have all the different groups in the original articles.
